# The role of pre-reduction MRI in the management of complex cervical spine fracture-dislocations: an ongoing controversy?

**DOI:** 10.1186/s13037-017-0139-8

**Published:** 2017-09-08

**Authors:** Sergiu Botolin, Todd F. VanderHeiden, Ernest E. Moore, Herbert Fried, Philip F. Stahel

**Affiliations:** 1 Department of Orthopaedics, University of Colorado, School of Medicine and Denver Health Medical Center, 777 Bannock Street, Denver, CO 80204 USA; 2 Department of Surgery, University of Colorado, School of Medicine and Denver Health Medical Center, Denver, CO 80204 USA; 3 Department of Neurosurgery, University of Colorado, School of Medicine and Denver Health Medical Center, 777 Bannock Street, Denver, CO 80204 USA

**Keywords:** Cervical spine trauma, Fracture-dislocation, Perched facet, Intervertebral disc herniation, Timing of surgery, Spinal cord injury, Patient safety

## Abstract

**Background:**

Cervical spine fracture-dislocations in neurologically intact patients represent a surgical challenge due to the risk of inflicting iatrogenic spinal cord compression by closed reduction maneuvers. The use of MRI for early advanced imaging in these injuries remains controversially debated.

**Case presentation:**

A 54-year old man sustained a fall over the handlebars of his racing bicycle. The helmeted patient sustained a fall on his head which resulted in a hyperflexion injury of the neck. He was neurologically intact on presentation. Initial CT imaging revealed a complex multisegmental cervical spine injury with a left-sided C6/C7 perched facet, a right sided C7/T1 fracture-dislocation, and a right-sided C6 and C7 traumatic laminotomy. The initial management consisted of temporary external Halo fixator application without closed reduction maneuver, to mitigate the risk of a delayed spinal cord injury. Subsequent advanced imaging by MRI revealed an acute traumatic C7/T1 disc herniation, with the intervertebral disc completely extruded into the spinal canal. Definitive surgical management was then accomplished by employing a three-stage anterior-posterior-anterior spinal decompression, realignment, fixation and fusion C4-T2 in one operative session. The patient recovered well and retained full neurological function. He resumed bicycle street racing within 10 months of the injury following successful spinal reconstruction.

**Conclusions:**

The diagnostic evaluation of cervical fracture-dislocations should include advanced imaging by MRI in order to fully understand the injury pattern prior to proceeding with spinal reduction maneuvers which may impose the imminent threat of a devastating iatrogenic injury to the spinal cord. The presented staged management by initial Halo fixation without attempts for spinal reduction, followed by a surgical decompression and multilevel fusion, appears to represent a feasible and safe strategy for patients at risk of a delayed neurological injury.

## Background

Cervical spine fracture-dislocations continue to represent a significant challenge in trauma patients due to the imminent risk of neurological deterioration associated with potentially inadequate timing and modality of surgical management [1–3]. Fractures or dislocations of the posterior cervical elements are typically managed by an attempt for initial closed reduction with temporary external fixation in a Halo vest, followed by definitive posterior spinal fusion, as indicated [4–7]. However, a “classic” challenge for the management of cervical facet dislocations is represented by the potential of an associated injury to the anterior spinal column with a disc herniation into the anterior spinal canal [8]. In this scenario, an imprudent closed reduction maneuver may lead to the iatrogenic compression of the spinal cord with the potential for subsequent devastating neurological consequences [9–12]. The option of obtaining advanced imaging by MRI prior to a closed reduction maneuver remains controversial [13, 14]. While MRI undoubtedly represents the most sensitive diagnostic tool to evaluate for associated disc herniation, ligament injury, and traumatic myelopathy [15, 16], concerns about the standard use of MRI in the work-up of cervical facet dislocations relate to the delayed timing of early spinal realignment, considerations related to cost effectiveness, resource utilization, and the restricted availability of MRI across the globe [13, 17, 18]. Impressively, early studies on the use of MRI in cervical spine injuries revealed a presence of traumatic disc herniation in more than 40% of all patients [8]. In absence of MRI, the concept of closed reduction of the cervical spine in awake and alert patients has been largely proven safe and feasible [19–21], yet, selected cases of catastrophic deterioration of the neurological status after closed reduction maneuvers have been reported [10, 14, 22, 23]. In certain instances of cervical fracture-dislocations, patients owe an intact neurologic status to the fracture of the posterior elements, such as pedicle or lamina fractures (so-called “saving” laminotomy), which result in increased spinal canal space and thus prevent a traumatic spinal cord compression [21, 24]. The definitive surgical management of cervical fracture-dislocations with associated traumatic disc herniation is achieved via anterior, posterior or combined (anterior-posterior and anterior-posterior-anterior) approaches [2, 25, 26], however, in the setting of a neurologically intact patient, there is a general consensus to start the procedure through an anterior approach for spinal canal decompression [6]. In the present case report, we present a rare injury pattern of a cervical spine fracture-dislocation with rotational instability, posterior perched facet, and complete anterior extrusion of the intervertebral disc in a young and active patient without associated spinal cord injury. A safe surgical management strategy is presented and placed into context of the peer-reviewed literature in the field.

## Case presentation

**Fig. 1:**
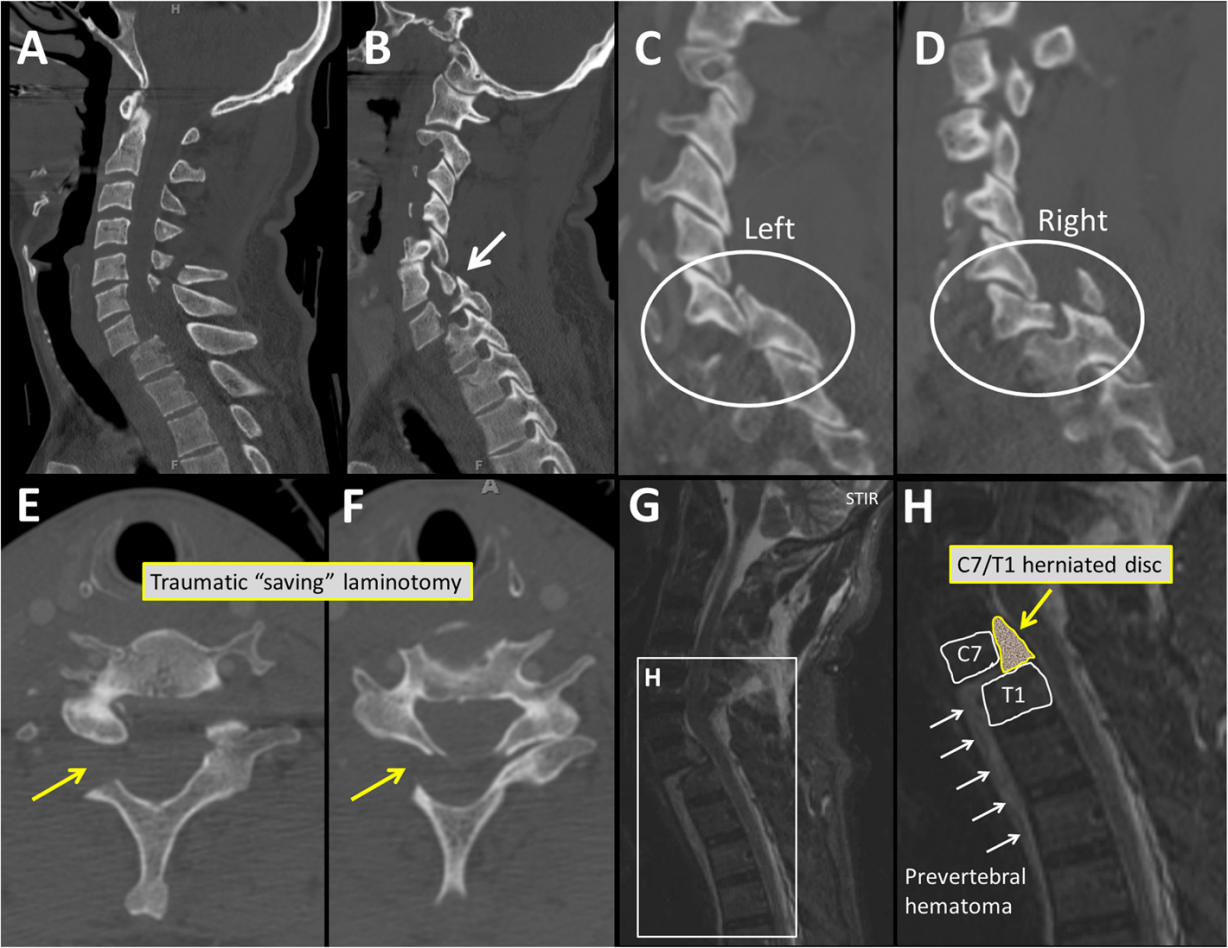
Initial diagnostic workup of the cervical spine injury with CT (panels **a**-**f**) and MRI (panels G,H). The sagittal views in panels **a**-**d** demonstrate the fracture dislocation with the left sided perched facet (panel **c**, and arrow in panel **b**) and the right sided facet fracture-dislocation (panel **d**). The posterior traumatic laminotomy is shown in axial CT images of C6 (panel **e**) and C7 (panel **f**). Panels **g** and **h** are sagittal STIR-weighed MRI images of the cervical spine, demonstrating the extruded C7/T1 disc within the spinal canal (panel **h** is a magnification of panel **g**

A 54-year old athletic male was a helmeted cyclist on a street race bicycle, when he fell over the handlebars and struck his head directly on cemented ground, sustaining a hyperflexion injury to his neck. He was able to get up at the accident scene and pushed his bicycle to the closest meeting point. Due to severe neck pain, he asked a friend to call an ambulance. He was initially brought to a local hospital in the Rocky Mountain region, where a CT of the head and cervical spine was obtained. The patient was placed in a C-collar and transported by ambulance to our level 1 trauma center for definitive management of his cervical spine fracture. Upon arrival, the patient was awake and alert with a GCS of 15. He stated to have felt some tingling sensation in his right hand after the accident, which apparently subsided by the time of his presentation to our hospital. His clinical exam demonstrated full symmetric motor strength (M5/5) in all four extremities, a normal rectal tone and bulbocavernosus reflex, and minimal hypoesthesia in the small finger on the right hand, related to the C8 dermatome. The review of his CT scan from the outside facility revealed a complex multi-level fracture-dislocation of the cervical spine from C6 to T1, with rotational instability (AO/OTA classification 51-C2.1). The injury pattern included a C7/T1 fracture-dislocation (Fig. 1a) with a left-side locked/perched facet at C6/C7 (Fig. 1b,c), a right side facet fracture-dislocation at C7/T1 (Fig. 1d), and a “saving” traumatic laminotomy at C6 and C7 on right side (Fig. 1e,f). A CT-angiogram was obtained which demonstrated a grade 1 intimal tear to the left vertebral artery at the C6 level. In light of the highly unstable fracture pattern, we decided to apply a Halo fixator for temporary external fixation. The Halo application was performed under local anesthesia without any closed reduction attempts, in order to mitigate the risk of an iatrogenic compression of the spinal cord. The patient was kept awake throughout and reported no change in symptoms. Thereafter, an MRI of the cervical spine was obtained for advanced imaging and preoperative planning. The MRI demonstrated a complete disruption of the annulus fibrosus of the C7/T1 intervertebral disc, a disruption of the anterior longitudinal ligament (ALL) and posterior longitudinal ligament (PLL) at the same level, and a complete extrusion of the C7/T1 disc into the spinal canal, positioned behind the C7 vertebral body (Fig. 1g,h). The interspinous ligament was disrupted at C6/C7, and there was mild spinal canal stenosis at C6/C7 without signs of cord compression or contusion.

A surgical plan was designed based on the following considerations:

1. The posterior perched facet requiring posterior approach and open reduction to restore anatomic sagittal alignment of the spine;
2. The extruded disc at C7/T1 requiring a C7 corpectomy and decompression through an anterior approach;
3. The sequence of surgery being dictated by the intraspinal disc which requires (1) anterior decompression, followed by (2) posterior fracture reduction, and ultimately (3) an anterior completion fusion after posterior reduction and instrumentation.

**Fig. 2:**
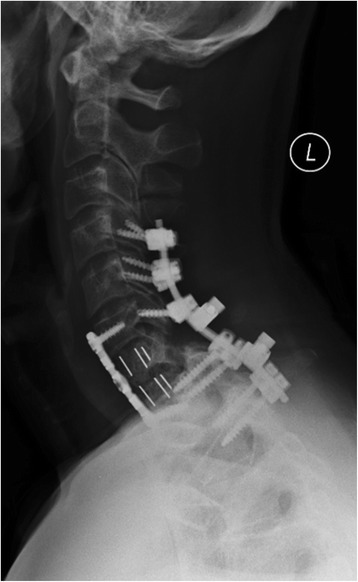
Lateral radiograph of the cervical spine after posterior C4-T1 fusion and anterior C7 corpectomy and fusion

**Fig. 3:**
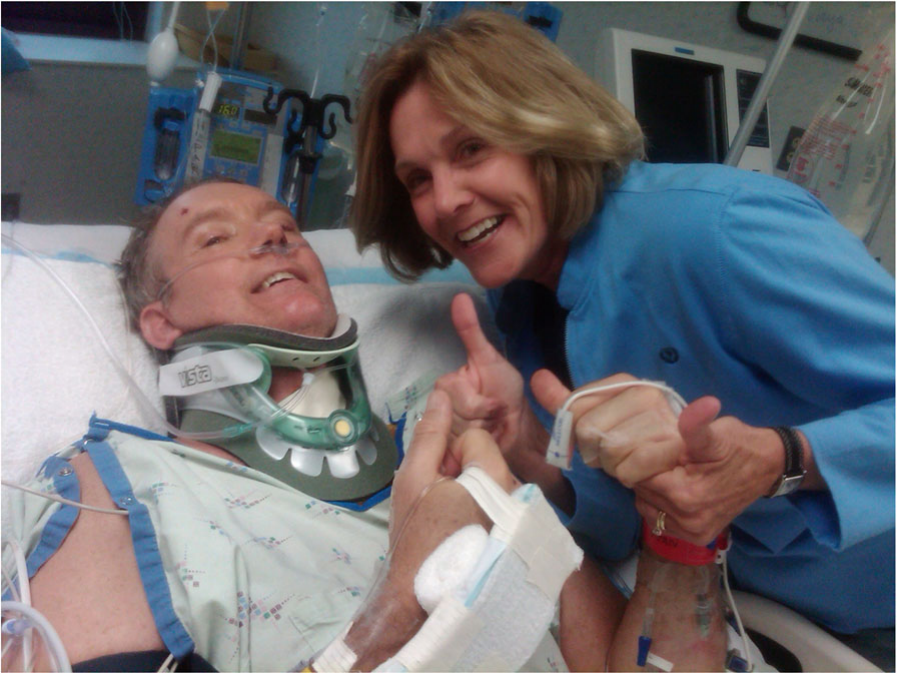
The patient and his wife giving a “thumbs up” in the surgical intensive care unit on postoperative day 1 after spinal fusion and Halo removal

**Fig. 4:**
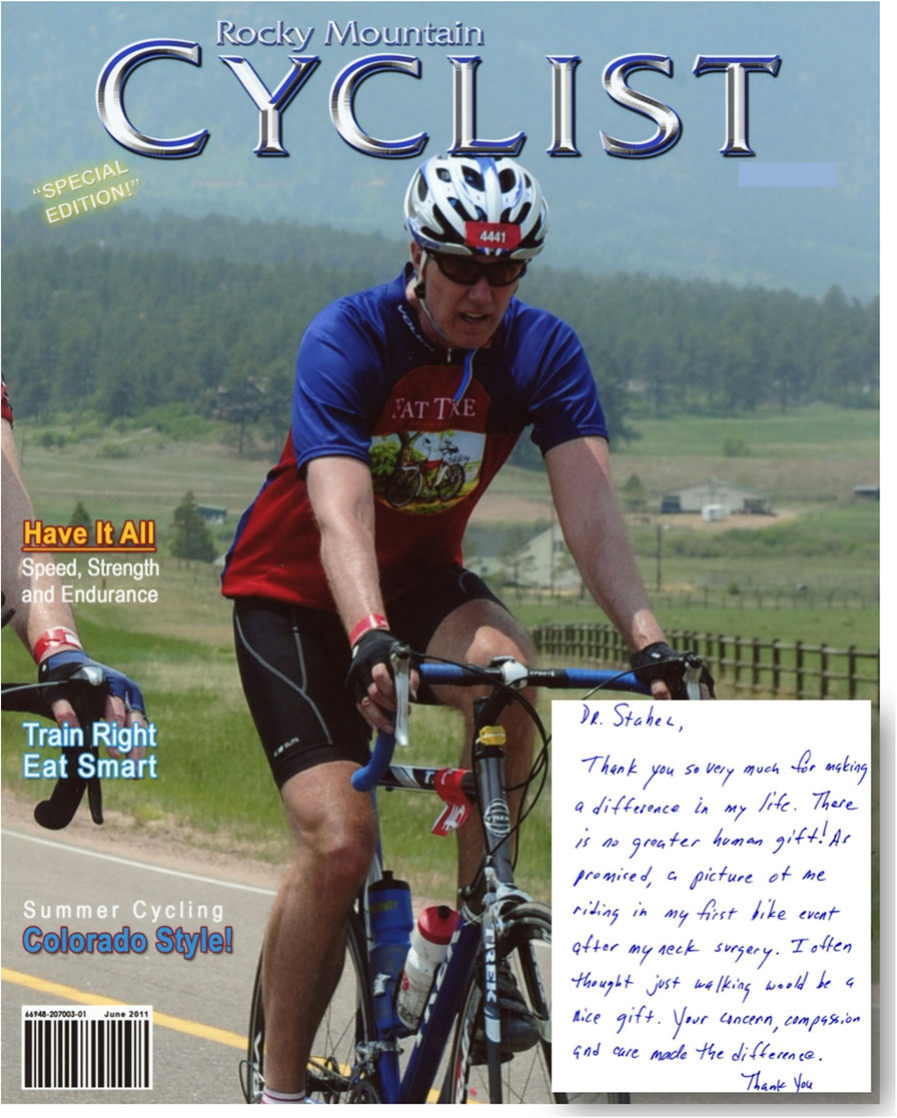
The patient depicted on his first street bicycle race at 10 months post injury, with an accompanying letter of gratitude

As part of the preoperative shared decision-making process for surgery, the patient understood the detailed plan and the risks of intra- and postoperative complications. He agreed to proceed with surgery and provided a written informed consent. We took him to the operating room the next day for the 3-stage procedure under continuous neurophysiological monitoring. In supine position, the cervical spine was exposed from C6-T1 through a standard left side anterior Smith-Robinson approach. The dislocation at C7/T1 was visualized and the intervertebral disc at this level was obliterated, as expected from the preoperative MRI findings. A C7 corpectomy was performed which allowed decompression of the spinal canal by removal of the extruded intervertebral disc. The anterior wound was then closed temporarily. Under strict log-roll precautions, using the Halo ring for axial traction, the patient was carefully moved into prone position, for the 2nd stage of the procedure. We then performed a standard posterior approach from C4-T2. A completion laminectomy was performed through the laminar fractures at C6 and C7, for posterior spinal canal decompression. The perched facet at C6/C7 on the left and the facet fracture-dislocation at C7/T1 on the right were anatomically reduced, and a left side C6/C7 facetectomy was performed, which resulted in adequate rotational and sagittal alignment of the cervical spine. Spinal fixation was performed by posterior instrumentation with placement of lateral mass screws at C4, C5, C6, and pedicle screws at T1 and T2. The posterior wound was closed, and the patient was log-rolled back into supine position for the 3rd stage of the procedure. The previous anterior cervical wound was re-opened. The exposed dura was visualized through the preceding C7 corpectomy, and a 2-level anterior fusion from C6-T1 was performed with a PEEK cage filled with autograft bone from the resected C7 vertebra, and placement of a 2-level anterior locking plate (Fig. 2). The anterior wound was then closed, the Halo was removed, and the patient was transitioned into a cervical collar. He tolerated the procedure well and was extubated in the operating room. His neurological exam was normal and the hypoesthesia in the right C8 dermatoma resolved postoperatively (Fig. 3). The grade 1 vertebral artery intimal injury was managed conservatively with 325 mg acetylsalicylic acid (Aspirin) for three months. The patient was mobilized with physical therapy and discharged home on the 2nd postoperative day. He remained in a cervical collar for 6 weeks and was allowed to remove the collar for showering only during that period of time. The patient resumed full activity at three months. He was back riding his bicycle at 7 months, and by 10 months after surgery he had returned to competitive street bicycle racing in the 100 mile “Elephant Rock Century Ride” in Castle Rock, Colorado (Fig. 4). The patient remained asymptomatic and continued his work as a financial consultant as well as unrestricted physical activity and exercises, including regular participation in competitive bicycle races, at follow up 7 years after the accident.

## Discussion

This report describes the rare case of a young active patient who sustained a complex cervical injury challenged by concomitant anterior disc protrusion and posterior facet dislocation in presence of intact neurological exam. This patient would have likely suffered a detrimental adverse neurological outcome if a standard closed reduction maneuver would have been performed in absence of MRI imaging. Even when performed under standard precautions in the awake patient, once the posterior perched facet jumps back into place with a conventional reduction maneuver by traction, hyperflexion and rotation, the large intraspinal disc fragment (Fig. 1g,h) would have likely led to an irreversible spinal cord compression with subsequent quadriplegia. The findings from the current case report on the successful management of a complex cervical spine fracture-dislocation without intra−/perioperative complications leading to an excellent long-term patient outcome provide a further argument towards the mandatory advanced imaging with MRI for the initial work-up of such risky traumatic conditions.

A prospective observational study by Vaccaro and colleagues from 1999 assessed the safety of awake closed reduction maneuvers in 11 patients with cervical spine dislocations who had a pre- and post-reduction MRI performed to assess for presence of intervertebral disc herniation [20]. Of these, 2 patients had disc herniations identified before reduction, 5 patients showed new disc herniations after the closed reduction maneuver, and 2 patients had irreducible dislocations [20]. While none of the patients in their study suffered from neurological worsening during or after closed reduction, these data raise some concern related to “blind” reduction maneuvers in absence of a re-reduction MRI. Suitably, Vaccaro stated in the conclusion of the article that the implications related to the *“neurologic safety of awake closed reduction traction reduction remains unclear”* [20]. Other studies on closed reduction of locked facets were performed in patients with complete or incomplete spinal cord injury [19], which represent a different entity compared the neurologically intact patient described in our case report, as it relates to the imminent risk of iatrogenic spinal cord compression leading to a preventable neurologic injury. Other groups have recommended MRI-guided reduction due to their observation of an incidence of 88% cervical disc disruption before closed reduction [27].

A similar case report as described here, on a neurologically intact patient with a C7/T1 cervical dislocation described the safe reduction in traction which was attributed to the associated fractures of the posterior spinal elements [21]. Arguably, the patient presented in our current case report retained his neurologically function due to the posterior “saving” traumatic laminotomy (Fig. 1e,f) which allowed for a temporary functional decompression of the spinal canal. The risk of a catastrophic neurological deterioration due to intervertebral disc herniation leading to iatrogenic cord compression during closed reduction of cervical spine dislocations is well documented in the peer-reviewed literature [9, 22].

In spite of the high risk of inducing preventable severe neurologic complications by inconsiderate reduction maneuvers, the timing and utilization of advanced diagnostics by MRI appears highly variable and remains contested [18]. Therefore, the conservative and cautious staged approach applied to our patient in the present case report appears justified and safe. First, we performed a preliminary stabilization of the cervical spine in a Halo vest with traction, which allowed monitoring the neurologic status in the awake patient until advanced imaging was obtained. The subsequent MRI indeed demonstrated a large posterior herniation of the entire C7/T1 disc into the spinal canal (Fig. 1g,h). Once diagnosis is established, different surgical treatment concepts have been described to mitigate the risk of intraoperative spinal cord compression in patients with cervical facet dislocations [6, 28–30]. Associated disc herniation appears to be treated by most spine surgeons through an anterior approach, either alone or in combination with a posterior approach for fracture reduction and stabilization/fusion [6]. In our case, we performed a three-stage anterior-posterior-anterior spinal decompression and C4-T2 spinal fusion; (1) in supine position, the herniated disc was removed through an anterior C7 corpectomy; (2) in prone position, a completion laminectomy of the traumatic laminotomy was performed prior to reduction of the locked facet on one side, and a facetectomy on the other side, and multilevel posterior instrumentation and fixation; (3) in supine position, a completion anterior fusion was performed. The anterior-posterior-anterior approach has been previously described and effectively applied in the management of complex cervical spine dislocations [26, 31]. A valid alternative consists of a posterior-anterior-posterior approach, which has been previously described in the following sequence; (1) a posterior approach for a complete facetectomy without attempts at fracture reduction; (2) an anterior discectomy with reduction of the dislocation and anterior fusion; and (3) posterior completion reduction and fixation/fusion [29]. Independent of the final surgical management strategy, we believe that the key to successful management of neurologically intact patients with cervical spine fracture-dislocations consists of early recognition of the presence of a herniated intervertebral disc prior to receding either with a temporary closed reduction or early definitive surgical decompression and stabilization.

## Conclusion

We strongly recommend obtaining advanced imaging with a pre-reduction MRI in neurologically intact patients with cervical spine dislocations for early recognition of an intraspinal herniated disc which places the spinal cord at risk for iatrogenic compression during closed reduction maneuvers, with the potential for catastrophic subsequent neurological complications.
